# Better Training for Matrons

**Published:** 1909-01-09

**Authors:** 


### Nursing Administration.

### BETTER TRAINING FOR MATRONS.

### I.—DEFECTS OF PRESENT SYSTEM.

The post of matron is growing every day wider in scope and more influential in character. It has been differentiated from the other kinds of nursing until it has developed into a highly specialised business. The main-spring of economy is the matron. She is the hinge on which order, efficiency, and comfort throughout the whole establishment may be said to turn. The training school rises or falls in public estimation in response to her endeavours, and the financial prosperity of the hospital is affected by her power of producing the right effect to an extent which wise managers know only too well. For all these complex responsibilities the matron gets as a general rule no further preparation than she has been able to procure by watching the course of events in the hospitals where she has been trained and has subsequently worked. True, in order to obtain any good post as matron, she will need good testimonials of success in departments of work nearly approximating to those of the matron. But there is commonly a conspicuous want of cohesion about knowledge acquired in this practical and somewhat casual manner. It must be said for it that it far outweighs any purely theoretical scheme of instruction, and that a few months spent as house-keeping sister in a well-managed institution will be of more service to a matron than a long course of study in a school for housekeepers. But although this is true, it by no means follows that the present system of seizing what opportunities she can and guessing the rest is an ideal one for nursing education generally and for the good of institutions depending so materially on the possession of a capable matron. It is time to recognise that whereas there are two distinct classes of women at present continually under training—the one as private and district nurses, and the other for administrative posts—the education which suffices for one group is far from adequate for the other. Failure to come to a clear understanding on this point is responsible for much overstrain and disappointment in the training school on the one hand, and, on the other, for a regrettably low standard. One school of thought in nursing matters lays continual stress on the supreme importance of sound practical work in the wards, and reduces theoretical knowledge to the minimum, contending that the nurse in private work is none the better for having gained diplomas and medals, and is better employed in exercises of manual efficiency. The other school multiplies tests of intellectual ability, and points to the triumphs of the nurse in the examination-room with justifiable pride. Both kinds undeniably make good nurses, but since in private practice the unlearned are admittedly of equal service with the more brilliantly endowed, would it not be wise to direct the energies of able pupils into channels where the knowledge they can acquire will advantage them in their future career? It can hardly be denied that nurses who are intensely absorbed in the scientific aspect of their work—in advanced physiology, for instance, in chemistry, and the materia medica—have mistaken their career. They ought to have surrendered themselves as medical students in all sincerity to the mastery of subjects demanding many year's close and undivided study. Women who think, women who desire to master whatever they undertake, and are capable of sustained study are urgently needed in the nursing world. It is sheer waste of good material to let them follow blind trails in the direction of spurious medical knowledge, only to find themselves sooner or later barred by a wall beyond which they are not able to pass. Meantime the special branches of knowledge essential to the formation of an efficient matron are as completely disregarded in the generality of training schools as though we were living in that novelists' world wherein the widowed heroine retires from society "to become the matron of a hospital."

In certain large hospitals—in Guy's, for instance, and St. Thomas's—facilities are offered to sisters who desire to take up hospital appointments, for acquiring an insight into the matron's duties. Such sisters—and at Guy's the privilege is extended to sisters trained in other institutions—are permitted to take a few months' experience in each department. The home department, the office, the housekeeping, the laundry, the linen-rooms—all these branches of work are studied from within. The training thus given is probably as nearly perfect as it is in the nature of training to be. But among the thousands of matrons on whom the education of the next generation of nurses depends, how few have enjoyed such or similar advantages? Not only are the opportunities few, but the standard of things which a matron ought to be taught is all nebulous and unfixed. There is nothing in the nature of a curriculum to which the nurse with administrative gifts can be directed, so that she can estimate her own deficiencies and take steps to repair them before accepting a post of this nature.

There is grave reason to fear that were a definitely high standard of theoretical training imposed by law on nurses in general, many excellent women who are now attracted to the work would be lost to it. But the nursing world had never more need than at the present moment to attract women of exceptional ability. Compared with the need for a recognised standard of education for matrons, reforms in the training of private nurses are of secondary importance. At present there is no encouragement to systematic preparation for these posts, which yet demand acquaintance with many branches of knowledge outside the training of the nurse. The career of the matron should be set as a mark of attainment to those who are able and willing to surrender themselves to an extra year or two of systematic study and investigation. It should not be reserved for those who are able to command sufficient pleasing testimonials, supported by local interest, to carry the day.

